# Protective effect of heat-inactivated *Mycobacterium bovis* applied intramuscularly is associated with enhanced lung immune response in caprine tuberculosis

**DOI:** 10.1186/s13567-025-01634-5

**Published:** 2025-11-04

**Authors:** Irene Agulló-Ros, Leonor Muñoz-Fernández, Álvaro Roy, Javier Bezos, Iker A. Sevilla, Inmaculada Moreno, Joseba Garrido, Antonio Rodríguez-Bertos, Mercedes Domínguez, Ramón Juste, Lucas Domínguez, Christian Gortázar, María A. Risalde

**Affiliations:** 1 Departamento de Anatomía y Anatomía Patológica Comparadas y Toxicología, Grupo de Investigación en Sanidad Animal y Zoonosis (GISAZ), UIC Zoonosis y Enfermedades Emergentes ENZOEM, Universidad de Córdoba, Córdoba, Spain; 2 Centro Nacional de Epidemiología, Instituto de Salud Carlos III, Majadahonda, Madrid, Spain; 3 VISAVET Health Surveillance Center, Universidad Complutense de Madrid, Madrid, Spain; 4 Departamento de Sanidad Animal, Facultad de Veterinaria, Universidad Complutense de Madrid, Madrid, Spain; 5 Animal Health Department, NEIKER-Instituto Vasco de Investigación y Desarrollo Agrario, Derio, Bizkaia Spain; 6 VISAVET Health Surveillance Center, Department Internal Medicine and Animal Surgery, Veterinary School, Universidad Complutense de Madrid, Madrid, Spain; 7 Unidad de Inmunología Microbiana, Centro Nacional de Microbiología, Instituto de Investigación Carlos III, Majadahonda, Madrid, Spain; 8 SaBio (Health and Biotechnology), Instituto de Investigación en Recursos Cinegéticos IREC (UCLM-CSIC), Ciudad Real, Spain; 9 CIBERINFEC, ISCIII - CIBER de Enfermedades Infecciosas, Instituto de Salud Carlos III, Madrid, Spain

**Keywords:** Animal tuberculosis, *Capra hircus*, HIMB, pulmonary immune response, vaccine

## Abstract

Caprine tuberculosis (TB) causes a zoonotic disease with significant economic and health implications. However, excluding some regions, goat herds are not subjected to official TB eradication programs. Implementing vaccination protocols for this species could provide a complementary and effective control strategy against TB. We assessed the protective efficacy and immune response associated with a heat-inactivated *Mycobacterium bovis* (*M*. *bovis*)-based immunostimulant (HIMB) applied intramuscularly against caprine pulmonary TB on 20 kid goats (10 immunized, 10 controls) naturally exposed to *M*. *caprae* infected goats for 10 months. TB-compatible lung lesions were assessed, alongside a local immune response analysis by immunohistochemistry of cell populations (Macrophages (MΦs), neutrophils, T, and B lymphocytes) and associated immune mediators (iNOS, TNF-α, IL-1α, IL-6, IFN-γ, TGF-β, IL-4). In the control group, 60% of the animals showed TB compatible lesions, compared with 40% of the immunized animals, which also showed a 78% reduction (*p* = 0.03) in the lesion severity score. Moreover, immunized animals showed a higher number of M1 MΦs (*p* = 0.03), producers of iNOS, as well as a higher expression of TNFα (*p* = 0.04) and IL-1α (*p* = 0.03). These mediators play a key role in the activation of a Th1-type cellular immune responses effective against mycobacteria, associated with a response of T lymphocytes expressing IFNγ, whose response was increased in the immunized group (*p* = 0.05). These results suggest that immunization with HIMB reduced the number and severity of TB-associated pulmonary lesions, which could be linked with an enhanced production of immune mediators with an essential role in the activation of MΦs with bactericidal functions.

## Introduction

Animal tuberculosis (TB) is a widespread worldwide zoonotic infectious disease caused by members of the *Mycobacterium tuberculosis* complex (MTBC), predominantly *Mycobacterium bovis* (*M*. *bovis*) and *M*. *caprae*. It imposes significant economic burdens on livestock farming globally and persists as a critical global health threat, with zoonotic TB cases re-emerging in some low-middle income countries [1, 2]. Goats (*Capra aegagrus hircus*) are recognized as a primary domestic reservoir of MTBC [3–5] and a potential source of infection for other species with close epidemiological links, including cattle [4], pigs [6], sheep [7], and even humans [8]. The significance of this species in the transmission and maintenance of MTBC has been underscored by the recent identification of several human TB cases caused by *M*. *caprae* linked to goat farms [8]. This poses a significant challenge to the eradication of zoonotic TB, highlighting the need for effective complementary control measures. European official eradication campaigns only target those goat flocks that coexist or have epidemiological links with bovine herds [9, 10]. Consequently, in Spain, which has the second-largest goat population in the European Union [11], some regions have implemented mandatory and voluntary control programs for this species [12]. The control of animal TB acquires significance in high prevalence regions and in low-middle income countries lacking eradication programmes [13]. Furthermore, test and cull schemes may be unacceptable in low-middle income countries [14]. Research has demonstrated that vaccination may be a crucial tool in endemic areas with no effective TB control programs. It effectively reduces clinical symptomatology, tuberculous lesions, the spread of mycobacteria on the farms [15, 16], and the economic burden on farmers and society [17].

Currently, the only commercially licensed vaccine against TB is Bacillus Calmette-Guérin (BCG), whose efficacy—though limited—has been widely demonstrated in humans and domestic and wild animal species [13]. This efficacy has also been observed in *M*. *tuberculosis* vaccine (MTBVAC) and its vaccine prototype (SO2), where administration in goats resulted in a reduction in the number and severity of TB lesions [18, 19]. Nevertheless, the use of live vaccines presents safety limitations, including potential persistence in animal tissues [20], risks to immunocompromised individuals [21], excretion into the environment through faeces if administered orally [22], or the need for cold chain maintenance [23].

Inactivated vaccines offer an interesting alternative to overcome these limitations. An immunostimulant based on heat-inactivated *M*. *bovis* (HIMB) was developed and used in 2011 [23], leading to reductions in TB lesion and culture scores in sheep [24], pigs [25], badger [26], red deer [27], or wild boar [23] comparable to those achieved with BCG. These results provide evidence of the potential of HIMB for the control and protection against TB. This vaccine was also evaluated in goats, showing a reduction in TB lesions and bacterial loads [27, 28]. HIMB immunization showed a 44% reduction in the severity of macroscopic tuberculous compatible lesions (TBLs) and a significant reduction in the number of pulmonary affected lobes, with lesions only detected in the caudal lobes [29].

However, a deeper understanding of the immunological mechanisms driving its effectiveness of this immunostimulant against TB has not been elucidated. Thus, the main objective of this study was to characterize the pulmonary immune response and assess the effectiveness induced by HIMB in goats naturally exposed to *M*. *caprae*.

## Materials and methods

### Study design

**Figure 1:**
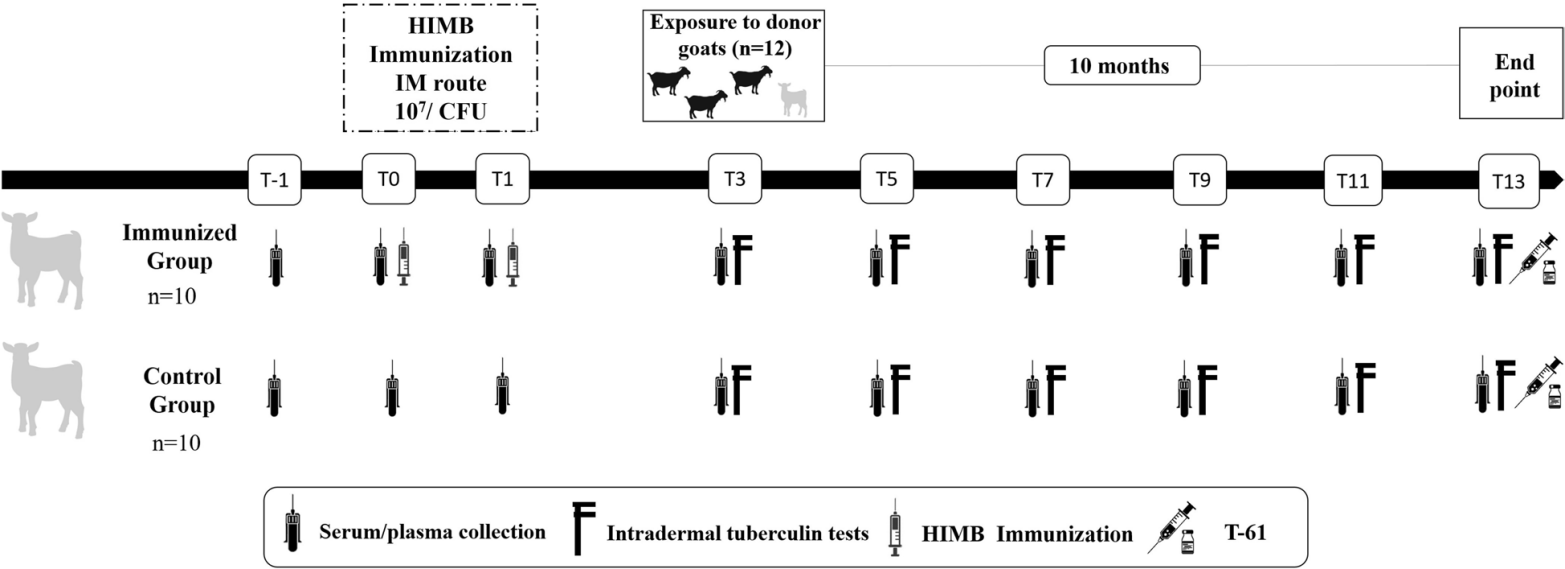
**Summary of the experimental design**. Silhouettes of grey kid goats represent the receptor animals, comprising both immunized with heat-inactivated *M*. *bovis* (HIMB) and control groups. The black goat silhouettes represent the donor goats infected with *M*. *caprae*.

The experimental design and preliminary results were widely described previously [29]. This study constitutes a direct continuation of that work, focusing on the histopathological evaluation of the local immune response at the pulmonary level following the long-term contact exposure. Briefly, 20 kid goats (2–3 weeks old) were selected from a farm with no history of TB and negative to cellular and humoral diagnostic tests, including the interferon gamma (IFNγ) release assay (IGRA) test (Bovigam, Thermo Fisher Scientific, Waltham, USA), and an in-house indirect enzyme-linked immunosorbent assay (ELISA) based on the immunoprotein complex P22 [29]. The goats were randomly divided into two groups: the immunized group (*n* = 10), receiving intramuscular administration of two doses (1 mL each) of HIMB at a 4-week interval (T0, first dose), and nonimmunized or control group (*n* = 10). Three months after the first immunization (T3), all goats were placed in direct contact with 12 adult donor goats (3–5 years old) for 10 months excreting *M*. *caprae* (spoligotype SB0157, confirmed by bacterial culture). The shedding of *M*. *caprae* by the donor goats was assessed by collecting oropharyngeal swabs, which were tested twice during the study—once before mixing the donor goats with the vaccinated and control goats, and again 10 weeks later. Although shedding is likely intermittent, positive results at both time points, together with the subsequent infection observed in the study animals, confirm that donor goats were infectious and capable of transmitting *M*. *caprae* throughout the experiment. During this period, various TB diagnostic tests (single intradermal tuberculin test (SIT), IGRA, and ELISA) were conducted every 2 months (T3, T5, T7, T9, T11, and T13) to monitor immune responsiveness in course of the infection, as previously described [29]. At 10 months post-exposure (T13), both immunized (*n* = 10) and nonimmunized (*n* = 10) animals were euthanized with T-61 (Intervet S.A., Salamanca, Spain) administered intravenously (Figure 1). Subsequently, all animals underwent a standardized and systematic necropsy for the assessment of TBLs in head lymph nodes (LNs), including mandibular and retropharyngeal LNs, lung and pulmonary LNs, liver, spleen, kidneys, hepatic, and mesenteric LNs. LNs and organs from other additional locations were assessed only if suspicious macroscopic TBLs were observed. A pooled sample of all the described organ tissues for each animal was collected in a sterile container and stored at −80 °C until bacteriological culture. Moreover, samples from each lung lobe for histopathology analysis were fixed in 10% neutral buffered formalin for 24–48 h, then dehydrated and embedded in paraffin using an automatic processor.

This study was conducted in accordance with European [30] and Spanish legislation [31]. It was approved by the Ethics Committee of the Complutense University of Madrid and the Regional Agriculture Authority of Comunidad de Madrid (permit number: PROEX 143/15, 29/06/2015).

### Preparation of HIMB vaccine

The HIMB immunostimulant consists of 10^7^ heat-inactivated colony-forming units (CFUs) of *M*. *bovis* from a naturally infected wild boar isolate (strain 1403; spoligotype SB0339), accompanied by the adjuvant Montanide ISA 50 V2 (Seppic, Castres, France). This immunostimulant was prepared following the protocol described previously [23], including a prolonged inactivation phase at 83 °C for 45 min [32].

### TB testing

Goats were tested every two months after booster vaccination using the SIT, IGRA, and an in-house ELISA, as previously described [29]. Briefly, the SIT was performed in the cervical region with bovine purified protein derivative (PPD) (CZ Veterinaria SA, Spain), and animals were considered positive if skin thickness increased ≥ 4 mm or clinical signs were observed 72 h post-injection (Royal Decree 2611/1996 and Directive 64/432/EEC). For the IGRA, heparinized blood was stimulated with bovine and avian PPD, and a sample was positive if the response to bovine PPD exceeded that to avian PPD by ≥ 0.05 OD. Antibodies against the MTBC were assessed using a modified ELISA [33], based on the immunopurified P22 subcomplex from bovine PPD, including MPB70, MPB83, ESAT-6, and CFP-10 [34]. Sera were tested in duplicate, and results were expressed as ELISA percentage (*E*%) = (mean sample OD/2 × mean negative control OD) × 100. Samples with *E*% > 100 were considered positive.

### Gross TB pathology in the lung

During necropsy, the scoring of TBLs in the lungs was based on two complementary systems that were subsequently combined. First, the presence, number, and size of TBLs were assessed using the scoring system described previously [29]: 0 = no visible lesions; 1 = no macroscopic lesions, but lesions evident on section; 2 =  ≤ 5 macroscopic lesions < 10 mm in diameter; 3 =  ≥ 6 macroscopic lesions < 10 mm in diameter or a single distinct macroscopic lesion > 10 mm in diameter; 4 =  > 1 distinct macroscopic lesion > 10 mm in diameter; 5 = coalescing macroscopic lesions. Second, the extent of the affected lung lobe was classified as follows: grade 0 = no visible lesions; grade I =  < 25% of the affected lobe with TBLs; grade II = 25–50%; grade III = 50–75%; and grade IV =  > 75%. This four-grade classification was adapted in the present study, modifying and applying a more detailed and standardized scoring system to quantitatively assess the extent and severity of pulmonary involvement. To calculate the total lung score, the values obtained from the two systems (number/size and extent of TBLs) were added. An additional point was assigned to animals with fibrous pleuritis.

### Histopathological evaluation of TBLs in the lung

The lung samples analyzed in this study originated from the experimental infection previously reported by Roy et al. [29]. In contrast to that previous work, the present study involved a detailed and systematic histopathological analysis of the pulmonary lesions. Specifically, all lung lobes were individually examined, and the granulomas identified were classified and scored according to their developmental stage and severity. This in-depth evaluation allows for a more refined assessment of the pathological impact of immunization with HIMB at the pulmonary level.

Lung samples fixed in formalin and Bouin’s solution underwent dehydration using an ascending alcohol ladder, followed by rinsing in xylene and were embedded in paraffin for preservation. A Leica TP1020 tissue processor (Wetzlar, Germany) and a Tissue-Tek® TEC® 5 paraffin dispenser (SakuraSeiki Co., Ltd., Japan) were employed. Histological sections with a thickness of 3–4 μm were prepared from the paraffin-embedded samples using a microtome (Microm® model HM325, Thermo Scientific™) for the histopathological study of TBLs through haematoxylin–eosin (H-E) staining. Macroscopic TBLs observed at the pulmonary level were histopathologically confirmed and differentiated according to a modified classification system from 2005 [35]. Distinctions were made between early stage or immature granulomas (stages I and II), characterised by the presence of epithelioid macrophages (MΦs) and variable infiltrates of lymphocytes, neutrophils and Langhans giant cells; and advanced stage or mature granulomas (stages III and IV), with the presence of extensive areas of necrosis or calcification. Subsequently, the microscopically assessed tuberculous granulomas received an additional numerical score based on their stage of development: 0 = no granulomatous lesions; 1 =  < 5 immature granulomas; 2 =  < 5 mature granulomas or between 5–10 immature granulomas; 3 = between 5–10 mature granulomas or > 10 immature granulomas; 4 =  > 10 mature granulomas. Evaluation of lesions was carried out by two experienced observers (I.A-R. and MA.R.) who were blinded to the group being examined.

### Characterization of pulmonary immune cells and mediators by immunohistochemistry (IHC)

Immunohistochemical staining of immune cell populations (M1 MΦs iNOS+, M2 MΦs, neutrophils, T and B lymphocytes) and immune mediators (TNFα, IL-1α, IL-6, IFNγ, TGF-β, and IL-4) was performed on histological sections obtained from the left cranial lobe and right caudal lobe of the lung in both groups owing to the high number of TBLs and the number of affected animals. We used the avidin–biotin-peroxidase complex (ABC) method with some modifications as previously described [36]. The tissue samples were deparaffinised and hydrated by three baths in xylene (10 min (min) each), a descending scale of alcohols (5 min each), a bath in distilled water (5 min) and one in phosphate buffer saline (PBS; 10 min) (pH 7.2). Endogenous peroxidase activity was depleted by incubation of the samples with 0.3% hydrogen peroxide in methanol for 30 min at room temperature (RT). The samples were subjected to different methods for antigen retrieval, depending on the primary antibody (Ab) used (Table 1). Subsequently, sections were rinsed three times in PBS (pH 7.2) for 10 min, covered with 1% normal horse serum (Pierce-Endogen, Woburn, USA) in 0.05 M Tris buffered saline (TBS) (pH 7.6) or 20% normal goat serum (Thermo Fisher Scientific, Massachusetts, USA) for 30 min at RT, depending on secondary Ab. The slices were then incubated with the corresponding primary Ab overnight at 4 °C. After primary incubation, slides were washed in PBS (three times, 5 min each), then incubated with the secondary Abs for 30 min at RT. Biotinylated horse anti-mouse IgG secondary Ab (Pierce-Endogen, Woburn, USA) diluted 1:200 in TBS containing 1% normal horse serum was used for primary mouse Abs, and biotinylated goat anti-rabbit IgG secondary Ab (Vector Laboratories, CA, USA) diluted 1:200 in PBS containing 10% normal goat serum for primary rabbit Abs. After three further 5 min washes in PBS, the samples were incubated with ABC complex (Vectastain® ABC Elite Kit, Vector Laboratories, CA, USA) for 1 h at RT. All tissue sections were rinsed in TBS and incubated with chromogen solution (NovaRED® Substrate Kit, Vector Laboratories). Finally, the slides were counterstained with Harris’ haematoxylin.

**Table 1:** **Details of the primary antibodies used in the immunohistochemical study**

Specificity	Antigen or cell detected	mAb/pAb	Dilution	Pre-treatment	Commercial origin
Anti-human lysozyme	Neutrophils	pAb	1:200	Proteinase K^a^	Agilent Dako
Anti-iNOS	M1 MΦs	pAb	1:100	TC-autoclave^b^	Millipore ™
Anti-human CD163	M2 MΦs	mAb	1:200	TC-autoclave^b^	Bio-Rad AbD Serotec
Anti-human CD3	T lymphocytes	pAb	1:100	TC-microwave^c^	Agilent Dako
Anti-human CD20	B lymphocytes	pAb	1:200	TC-autoclave^b^	Bio-Rad AbD Serotec
Anti-bovine TNFα	TNFα	pAb	1:25	TC-oven^d^	Bio-Rad AbD Serotec
Anti-human IL-1α	IL-1	pAb	1:100	Tween 20^e^	Endogen
Anti-sheep IL-6	IL-6	mAb	1:50	TC-microwave^f^	Bio-Rad AbD Serotec
Anti-bovine INFγ	INFγ	mAb	1:10	TC-microwave^f^	Bio-Rad AbD Serotec
Anti-TGF-β	TGF-β	mAb	1:50	TC-microwave^f^	Bio-Rad AbD Serotec
Anti-bovine IL-4	IL-4	mAb	1:20	TC-microwave^b^	Bio-Rad AbD Serotec

mAb: monoclonal antibody; pAb: policlonal antibody.

^a^Incubation with 0.2% proteinase K (Sigma-Aldrich Chemie, Buchs, Switzerland) in Tris buffer for 8 min (min) at 37 °C in oven.

^b^Incubation with 0.1 M tri-sodium citrate dihydrate (pH6), autoclaved for 10 min at 121 °C and 1 atm.

^c^Incubation with 0.1 M tri-sodium citrate dihydrate (pH 6), microwave for 6 min at sub-boiling temperature.

^d^Incubation with 0.1 M tri-sodium citrate dihydrate (pH 6), oven for 30 min at 37 °C.

^e^ Incubation with Tween 20 for 30 min at room temperature.

^f^ Incubation with 0.1 M tri-sodium citrate dihydrate (pH 3.2), microwave for 6 min at sub-boiling temperature.

The immunolabeled cells were identified by IHC based on their location, size and morphological characteristics. Cell counts were conducted in 25 fields of 0.2 mm^2^ by two experienced observers (IA-R and LM-F) using random diagonal lines, and the results were expressed as cells per 0.2 mm^2^.

### Statistical analysis

The prevalence of macroscopic and microscopic TBLs in lung was estimated as the ratio of animals with TBLs to the total number of animals tested in the corresponding group, along with 95% confidence intervals (95% CI). Statistical analysis was conducted using the jamovi interface for R [37]. The normality of the distribution for macroscopic and histopathological lung lesion score data, as well as the expression of M1 MΦs iNOS+, M2 MΦs, neutrophils, T and B lymphocytes, TNFα, IL-1α, IL-6, IFNγ, TGF-β, and IL-4 in the left cranial lobe and right caudal lobe of the organ, was analyzed using the Kolmogorov–Smirnov test and was accepted when at least one group had a *p* value higher than 0.05.

As nearly all variables, except IL-6 and B lymphocytes, complied with this criterion, the data were submitted to a generalized linear model with post hoc pairwise comparison of the immunization groups with the Tukey test. Associations between variables were explored with a Principal Component Analysis (PCA) and further verified with a pairwise correlation analysis using both Pearson and Spearman tests to assess association strength and statistical significance [38]. Fisher’s exact test or the chi-square test (χ2) was also applied to analyze differences in TB granuloma presentation between immunized and control groups. T-values with a *p* < 0.05 were declared statistically significant.

## Results

### Antemortem evaluation

None of the animals included in the study showed clinical signs of TB (e.g., coughing, weight loss), although vaccinated kids exhibited a mild transient limp for several weeks post-vaccination. The results of diagnostic tests throughout the study are extensively reported and discussed in Roy et al. [29]. Briefly, all kid goats were negative to IGRA, ELISA, and SIT at T0. Then, 3 months after vaccination (T3), 80% of vaccinated animals were IGRA and SIT positive, while controls remained negative. At least 60% of vaccinated goats stayed positive until the end of the study, whereas controls only reacted from T9 after natural exposure. ELISA responses also appeared earlier in the vaccinated group (from T5) and remained significantly higher than in controls at almost all time points.

### HIMB reduces pulmonary lesions in goats naturally infected with *M*. *caprae*

**Figure 2:**
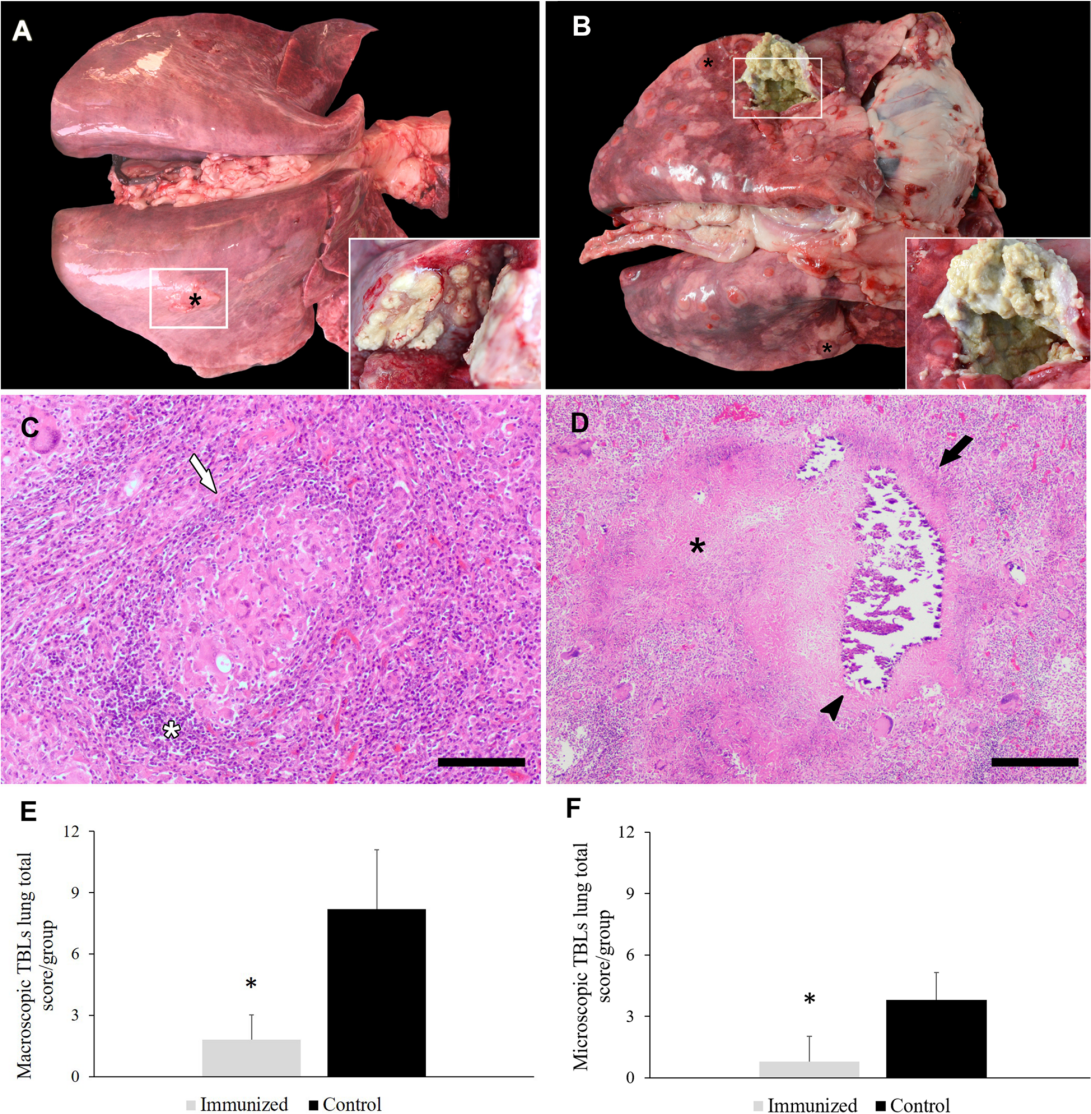
**Representative images of macroscopic tuberculous compatible lesions (black asterisks) (TBLs) of goats immunized with heat-inactivated *M. bovis*** **(HIMB) (A) and control (B) goats naturally infected with *M***. ***caprae***. Insets show close-up images at higher magnification of TBLs. Representative images of microscopic TBLs of immunized (**C**) and control (**D**) animals naturally infected with *M*. *caprae*. **C** Lung parenchyma of a goat immunized with heat-inactivated *M*. *bovis* (HIMB) showing microscopically an immature granuloma (white arrow) composed mainly of epithelioid MΦs and Langhans giant cells, surrounded by a mononuclear inflammatory infiltrate (white asterisk). Scale bar = 100 μm. **D** Multifocal mature granuloma (black arrow) in the lung of a nonimmunized animal, with several calcification foci (black arrowhead) and extensive areas of necrosis (black asterisk). Scale bar = 200 μm. Staining: haematoxylin–eosin. Mean ± standard error of the macroscopic (**E**) and microscopic (**F**) total lung scores of goats immunized with HIMB and controls*.* *p ≤ 0.05; Tukey test.

Following a 10-month exposure to *M*. *caprae* from donor goats and confirmed TB infection in both HIMB-immunized and nonimmunized goats, four animals (40%) in the immunized group exhibited macroscopic TBLs in lungs. These lesions were limited to the caudal lobes, occasionally encapsulated and with moderate caseous exudate (Figure 2A). By contrast, in the nonimmunized control group, TBLs were present in six goats (60%), with lesions observed in various lung lobes (five goats in the left cranial, four in the right cranial, four in the middle lobe, two in the accessory lobe, six in the left caudal, and five in the right caudal lobe). These lesions exhibited abundant caseous exudate in some cavernous-type lesions, with the presence of complete or incomplete capsules (Figure 2B). Furthermore, fibrous pleuritis was observed in 6/10 control animals (60%) compared with only 1/10 vaccinated animals (10%). Assessment of the mean scores in both groups, including animals with and without TBLs, revealed that HIMB-immunized animals had a score of 1.8 and an extension of less than 25% in the affected lobes. In contrast, the nonimmunized control group presented a mean score of 8.20, with lesions occupying between 50 and 75% of the lung parenchyma. Although no statistically significant differences were detected between both groups in the percentage of animals with TBLs in the lung (χ2 = 0.8, *p* = 0.37), immunized animals displayed a remarkable 78% reduction in the lesion severity score compared with the control group (*p* = 0.01) (Figure 2E).

The macroscopic TBLs in the lungs were histopathologically confirmed and assessed based on the previously established classification [35]. Microscopically, HIMB-immunized animals exhibited TBLs in the right and left caudal lobes, primarily displaying immature non-necrotic granulomas (Figure 2C). In contrast, the nonimmunized control group showed granulomatous lesions in all lung lobes, with a higher presence of mature necrotic granulomas (Figure 2D). In this context, immunized animals demonstrated a mean score of 0.80 compared with 3.80 observed in nonimmunized animals. Like in the case of macroscopic TBLs, no statistically significant differences were noted between both groups in the percentage of animals presenting granulomas in lung samples (*χ*2 = 0.8, *p* = 0.37). Nevertheless, a 79% reduction in the severity score of microscopic lung TBLs was recorded in immunized animals compared with the nonimmunized group (*p* = 0.03) (Figure 2F).

### HIMB stimulates a robust T lymphocyte response in goats naturally infected with *M*. *caprae*

**Figure 3:**
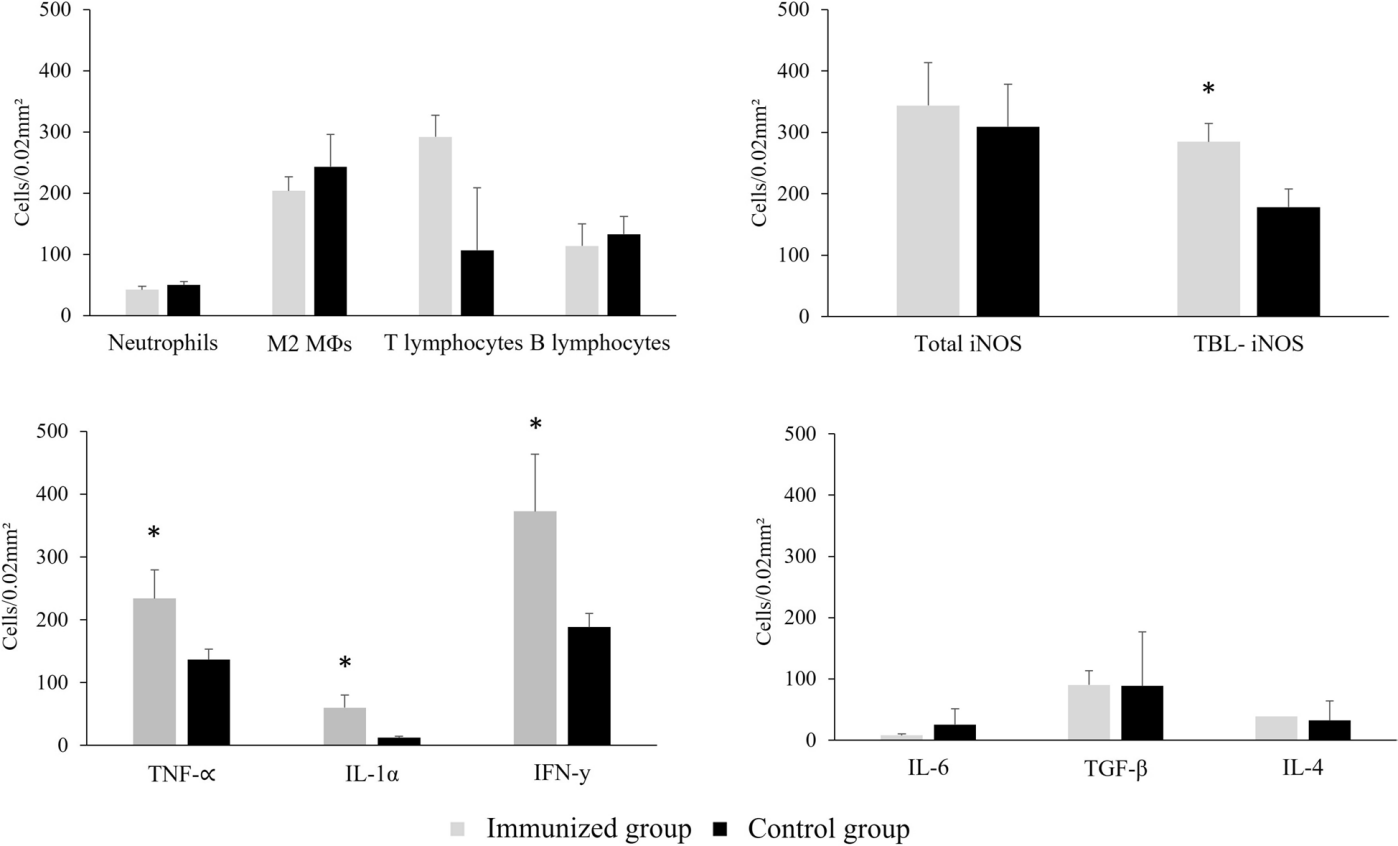
**Quantitative analysis (mean ± standard error) of various immune cells and immunological markers in the lungs of goats immunized and nonimmunized with heat-inactivated** ***M***. ***bovis*** **(HIMB) and exposed to** ***M***. ***caprae***. **A** Immunopositive neutrophils (lysozyme), M2 MΦs (CD163 +), T lymphocytes (CD3+), and B lymphocytes (CD79αcy+). **B** MΦs expressing iNOS evaluated in lung parenchyma including tuberculous granulomas (Total), and in lung parenchyma without lesions (TBL-). **C** Immune cells positive to TNFα, IL-1α and IFNγ. **D** Immune cells positive for IL-6, TGF-β and IL-4. *p ≤ 0.05: Tukey test.

The cell populations crucial for orchestrating the immune response to mycobacteria and participating in granuloma formation were evaluated. Neutrophils, M2 MΦs and B lymphocytes exhibited comparable counts in both groups of animals (Figure 3A), with M2 MΦs being the predominant population. In contrast, T lymphocytes displayed markedly elevated counts in the immunized goats (Figure 3A). Staining for neutrophils, identified by the lysozyme marker, was evident in the cytoplasm of scattered granulocyte-like cells in early granulomas (stages I and II), in necrotic areas and peripheral inflammatory infiltrates of advanced-stage granulomas (stages III and IV), as well as in the lung parenchyma without TBLs of both groups. M2 MΦs were identified by the CD163 marker, exhibiting granular cytoplasmic staining. This staining was mainly detected in nonimmunized animals around necrotic/mineralized areas of advanced granulomas, showing a diffuse distribution within the lung parenchyma and slightly lower numbers in immunized goats (Figure 3A). Indeed, a strong positive correlation was detected between these cells and microscopic TBLs (> 0.86; *p* < 0.001). T lymphocyte staining was observed in the cytoplasm and cell membrane of these cells using the CD3+ marker. This staining was predominantly concentrated in the lung parenchyma without TBLs of immunized animals, exhibiting significantly higher counts of these cells (*p* = 0.004) (Figure 3A). In nonimmunized animals, interspersed T lymphocytes were reported in initial stages and located at the outermost periphery of mature necrotic granulomas. B lymphocytes, identified by the CD79αcy + marker, exhibited a cytoplasmic staining pattern. These cells showed slightly higher counts in nonimmunized animals (Figure 3A), with distributions ranging from scattered in early stages to localized at the periphery of advanced granulomas. Additionally, B lymphocytes were observed to a lesser extent in lung parenchyma without TBLs.

### HIMB enhances activity of M1 MΦs with bactericidal function in goats naturally infected with *M*. *caprae*

**Figure 4:**
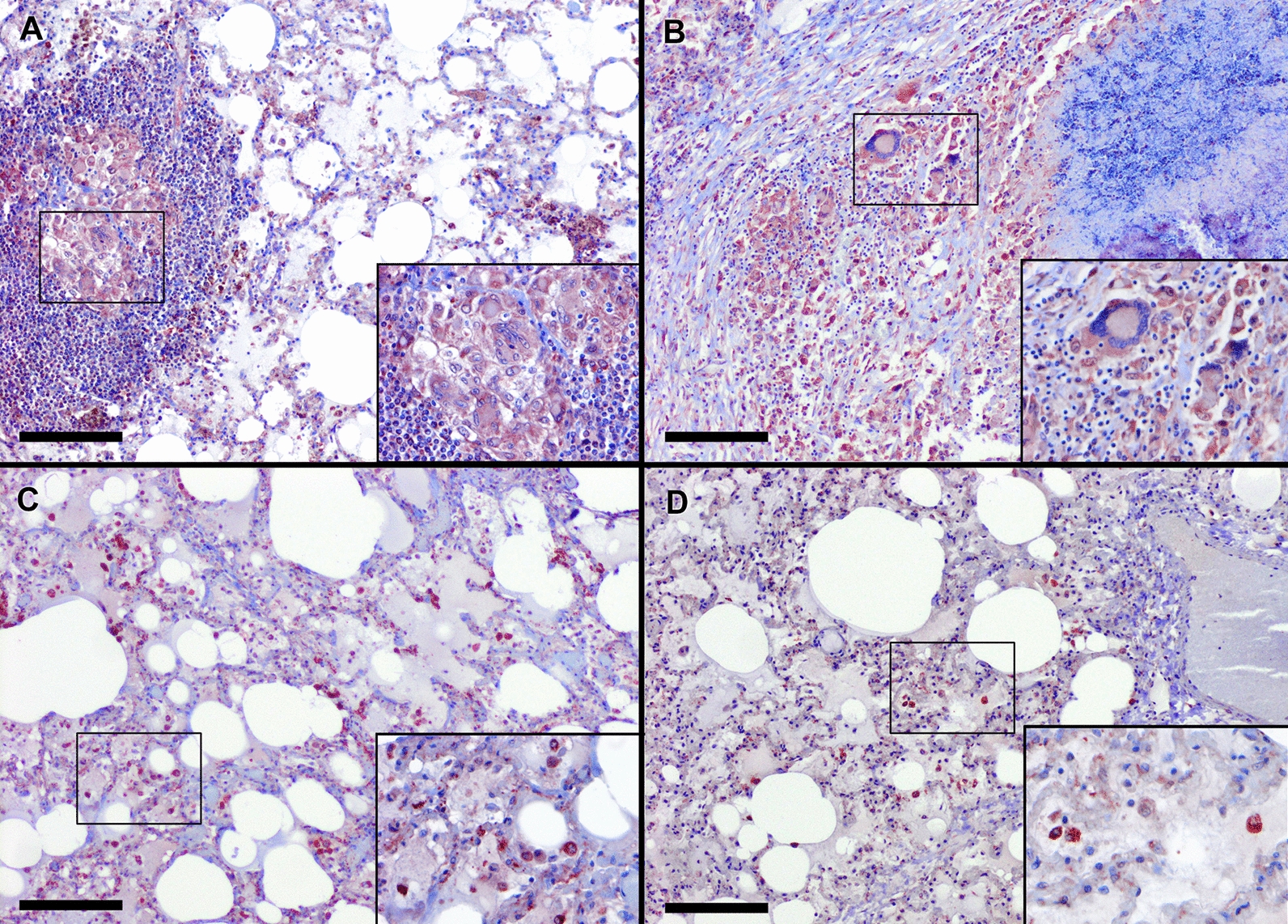
**iNOS expression in granulomatous and nongranulomatous lesions of goats immunized with HIMB and nonimmunized controls**. **A** Epithelioid MΦs (MΦs) and Langhans giant cells expressing iNOS in immature granulomas of goats immunized with heat-inactivated *M*. *bovis* (HIMB). **B** Epithelioid MΦs expressing iNOS at the periphery of mature granulomas in nonimmunized goats. **C** Diffuse alveolar and interstitial MΦs in lung parenchyma without lesions of goats immunized with HIMB. **D** Multifocal interstitial MΦs in lung parenchyma without lesions of nonimmunized goats. Insets show close-up images at higher magnification of MΦs iNOS+. Scale bar = 100 μm.

Alveolar and interstitial MΦs, together with epithelioid and Langhans giant cells, showed granular cytoplasmic iNOS immunostaining. In immunized animals, staining was mainly localized in initial TBLs (Figure 4A) and in the diffuse inflammatory infiltrate of the lung parenchyma without granulomas (Figure 4C). In contrast, in nonimmunized goats, iNOS+ MΦs were predominantly found at the periphery of mature necrotic granulomas (Figure 4B) and were scarce in nongranulomatous areas of the parenchyma (Figure 4D). Overall, the highest expression of this marker was associated with granulomas, which were considerably less numerous in the immunized animals. Although group differences in total lung iNOS+ expression (including TBLs) did not reach statistical significance (Figure 3B), in nonimmunized goats this marker correlated strongly with both gross (*r* > 0.81, *p* = 0.006) and microscopic lesion scores (*r* > 0.77, *p* = 0.010). In contrast, no correlation was found in immunized animals (gross: *r* = 0.19, *p* = 0.583; microscopic: *r* = 0.30, *p* = 0.386).

Interestingly, when analysis was restricted to lung areas without TBLs (TBL-iNOS), immunized goats showed significantly higher iNOS expression than controls (*p* = 0.03) (Figure 3B). In nonimmunized goats, this marker correlated strongly with lesion severity (gross: *r* > 0.86, *p* = 0.002; microscopic: *r* > 0.91, *p* = 0.001), whereas no correlation was observed in immunized animals (gross: *r* = 0.08, *p* = 0.811; microscopic: *r* = 0.18, *p* = 0.607).

### HIMB immunization stimulates a Th1 immune response in the lung of goats naturally infected with *M*. *caprae*

**Figure 5:**
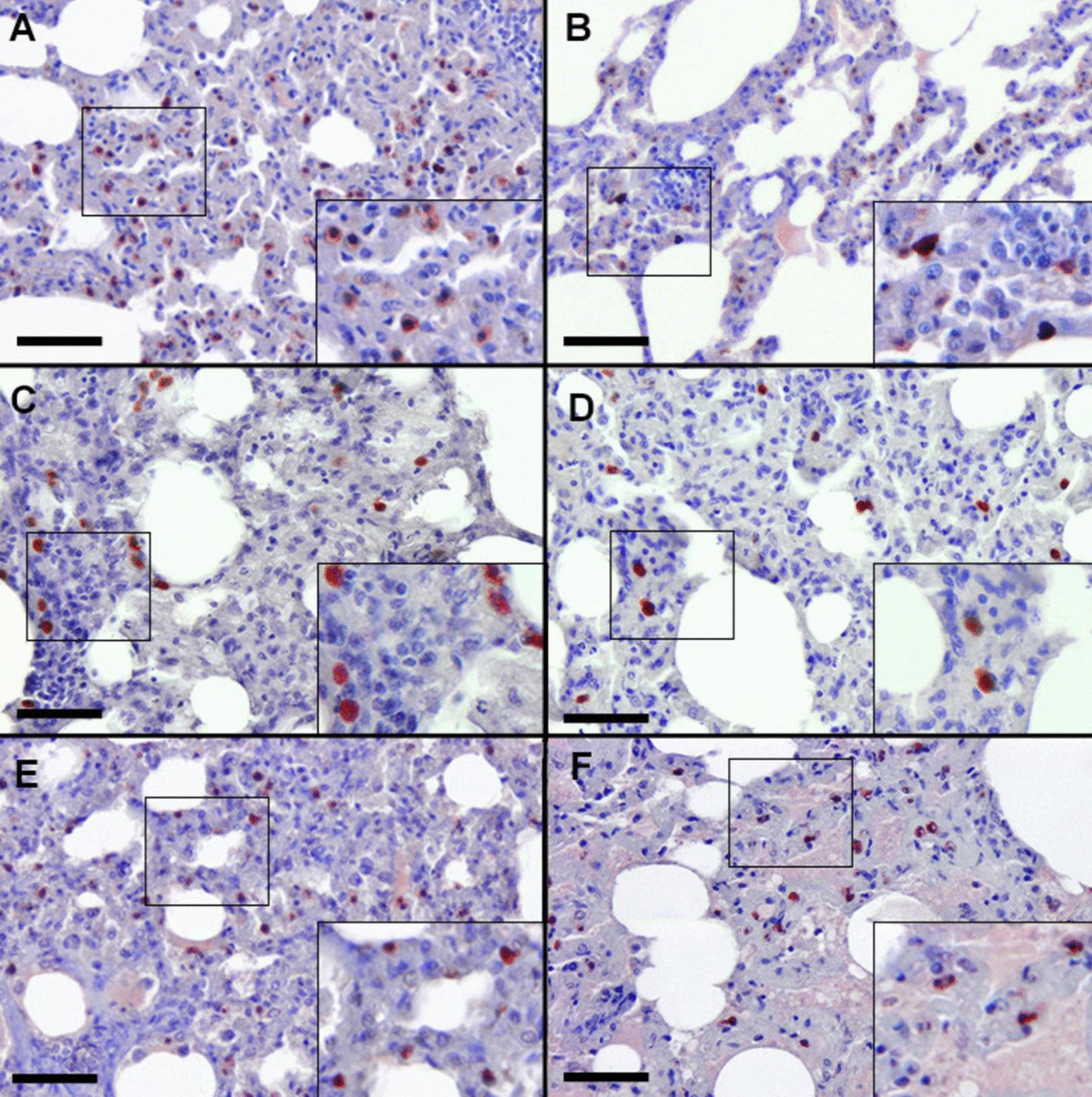
High expression of numerous cells positively stained for TNF-α (A), IL-1α (C) and IFNγ (E) in the lung parenchyma of goats immunized with heat-inactivated *M. bovis* (HIMB) compared with the expression of immune cells positively stained for TNFα (B), IL-1α (D) and IFNγ (F) in the lung parenchyma of nonimmunized goats. Insets show close-up images at higher magnification of TNFα, IL-1α, and IFNγ+ cells. Scale bar = 100 μm.

In the lung, the expression of pro-inflammatory cytokines TNFα and IL-1α, characterized by granular cytoplasmic staining, was associated with interstitial MΦs and neutrophils, distributed predominantly randomly throughout the lung parenchyma (Figures 5A–D), with heightened detection in immunized animals (*p* = 0.04 and 0.03, respectively; Figure 3C). Their presence in necrotic and non-necrotic granulomas was limited. IFNγ, a Th1-inducible cytokine, primarily exhibited cytoplasmic staining in lymphocytes of the pulmonary inflammatory infiltrate (Figures 5E, F), displaying greater expression in immunized goats (*p* = 0.03; Figure 3C). To a lesser extent, IFNγ was localised at the periphery of mature granulomas in control goats. Additionally, a positive correlation between this cytokine and T lymphocytes were reported (>0.56; *p* < 0.05).

IL-6 detection, characterized by cytoplasmic staining with a granular pattern, was observed in lower counts within interstitial MΦs and neutrophils, randomly distributed in the lung parenchyma and, to a lesser extent, in mature necrotic granulomas. Similarly, the expression of TGF-β in M2 MΦs and neutrophils, and the expression of IL-4 in lymphocytes, was observed diffusely in the lung parenchyma, with limited presence in immature and mature granulomas. There were no significant differences in the expression of these cytokines between HIMB-immunized and nonimmunized goats (Figure 3D). However, a moderate positive correlation was reported between TGF-β and M2 MΦs (> 0.53; *p* < 0.05) and neutrophils (> 0.49; *p* < 0.05).

## Discussion

This study marks the first comprehensive evaluation of immune responsiveness at the pulmonary level following intramuscular administration of an immunostimulant in domestic ruminants naturally infected with *M*. *caprae*. Our findings underscore the potential efficacy of HIMB in controlling goat TB, as evidenced by a notable reduction in the number, extent and severity of pulmonary TBLs in immunized animals. This reduction was associated with establishment of a Th1-type protective immune response induced by effector immune cells with an important microbicidal role. Moreover, a significant strength of this study is its realistic approach, as it was conducted under natural exposure conditions, despite the potential heterogeneity in results owing to the inability to control the time of infection and the infective dose [18, 19]. These results also highlight the potential benefit of immunoprophylaxis at an early age to induce protective immunity before natural exposure occurs, which could further enhance the control of TB in goat herds.

In our study, HIMB immunization reduced the pulmonary TBL severity score by at least 78% compared with nonimmunized animals. Lesions in immunized goats were predominantly small, non-necrotic, immature granulomas, mainly restricted to the caudal lobes, suggesting a vaccine-induced modulation of disease progression. This more contained infection could limit mycobacterial dissemination and transmission, thereby alleviate clinical signs and reduce environmental excretion [26, 39]. These findings highlight the potential of HIMB to reduce TBL severity and limit the spread of disease within the lungs and the environment. These results align with those reported by other authors in goats immunized with BCG, where immunization neither prevented infection nor allowed high severity of TBLs and bacterial load, limiting mycobacterial spread [40–42]. Moreover, following HIMB administration, a decrease in the incidence of goats with extrapulmonary TB had already been demonstrated, surpassing that observed with BCG or MTBVAC [19, 28]. Additionally, the efficacy of HIMB immunization in controlling severe TBLs and mycobacterial burden has been demonstrated in several domestic and wild species, resulting in decreased severity of clinical forms of the disease [23, 25–27, 43, 44].

The protective response against mycobacteria involves the activation of MΦs, DCs, NK cells and T cells [45]. Our findings underscore the effectiveness of HIMB immunization in establishing a protective Th1-type immune response against pulmonary TB, characterized by a significant increase of T lymphocytes and M1 MΦs populations, pivotal in producing key immunological mediators essential for controlling mycobacterial infections [46]. In our study, a high expression of iNOS, an essential compound for the nitric oxide production, was observed in M1 MΦs of immunized animals without TBLs or with mild lesions. Interestingly, while a strong positive correlation between iNOS expression and lesion severity was found in nonimmunized goats, no such correlation was observed in immunized animals. This suggests that, in vaccinated goats, the elevated presence of iNOS+ MΦs in nonlesioned lung areas may reflect an early and effective immune response that prevents lesion formation, thereby limiting the progression and severity of pulmonary TBLs in the context of HIMB-induced immunity. These results are consistent with the outcomes observed following BCG vaccination in cattle [47, 48]. Furthermore, TNFα and IL-1α play crucial roles in inducing chemokines and recruiting MΦs and T cells necessary for eliminating intracellular bacterial infections and maintaining the protective microenvironment of tuberculous granulomas [49, 50]. Our findings show an elevated expression of TNFα in immunized animals, consistent with observations in BCG-vaccinated cattle [47]. In addition, higher IL-1α expression, together with increased T lymphocyte counts, could explain the stronger IFN-γ response in this group, as IL-1α is a key mediator that enhances T cell activation and promotes IFN-γ production, as previously reported in *M*. *bovis*-challenged wild boar immunized with HIMB [25]. This cytokine plays a crucial role in host defense against intracellular infections [51] and was associated with the higher expression of iNOS+, a potent enzyme with bactericidal activity, and a pro-inflammatory cytokine response involved in mycobacterial control [52]. Taken together, our findings suggest that immunization with HIMB effectively triggers an adequate Th1 immune response against mycobacteria [47, 53].

Conversely, IL-6 expression was low in both nonimmunized and HIMB-immunized goats infected with *M*. *caprae*, unlike what is documented in BCG-immunized mice [54]. Supporting our findings about the relationship between IL-6 and Th-1 cytokines, several studies indicate that IL-6 interferes in the response of MΦs infected with mycobacteria to produce IFNγ, thus limiting the Th1 response’s ability to eradicate the infection [55, 56]. Similarly, the expression of anti-inflammatory cytokines that induce M2 MΦs and promote Th2 immune response development, such as TGF-β and IL-4, was severely limited in both groups, with a slight predominance in HIMB-immunized animals. These findings are consistent with the effects of heat-inactivated autovaccines against caprine TB [57] and BCG vaccination in other species, including bovines [58] and guinea pigs [59], indicating the maintenance of the pro-inflammatory response in combination with a suppressed anti-inflammatory response [58]. Conversely, control animals presented higher counts of M2 MΦs, highlighting a strong positive correlation with the presence of TBLs. This finding aligns with the anti-inflammatory tendency observed by other authors in the cellular populations surrounding the granuloma necrotic core [60].

In conclusion, intramuscular immunization with HIMB in goats resulted in a significant reduction of lung TBLs, associated with the induction of an effective local Th1 response. The decrease in the prevalence and severity of TBLs, particularly necrotic lesions, plays a significant role in diminishing mycobacterial burden and subsequent environmental excretion [61]. These findings establish a foundation for evaluating the efficacy of HIMB in field studies with larger animal cohorts to validate the observed findings and trends in reducing the severity of TBLs. Moreover, our study suggests the potential use of HIMB immunization in countries with a high prevalence of animal TB [62, 63], offering an alternative strategy to reducing TB rates [64]. Additionally, its application could be advantageous in low-middle income countries where TB control programs based on the diagnosis and slaughter of positive animals are not feasible.

## Data Availability

No datasets were generated or analyzed during the current study.
